# Decoding spontaneous intracerebral hemorrhage: mechanistic breakthroughs and disruptive revolution in pharmacological treatment

**DOI:** 10.1038/s12276-026-01733-z

**Published:** 2026-05-28

**Authors:** Yisheng Chen, Guanghui Wu, Wangzheqi Zhang, Zui Zou, Lei Huang, Haojun Shi, Qiangqiang Wang, Weijian Chen, Zhiwen Luo, Zhijie Zhao, Li Wu, Zhiwei Li, Jianhua Peng, Yujie Chen, John H. Zhang

**Affiliations:** 1 Fujian Key Laboratory of Toxicant and Drug Toxicology, undefined, Department of Vascular and Interventional Radiology, Ningde Municipal Hospital of Fujian Medical University, Ningde Normal University, Ningde, China; 2 Department of Neurosurgery, School of Medicine, Loma Linda University, Loma Linda, CA USA; 3 Department of Physiology and Pharmacology, School of Medicine, Loma Linda University, Loma Linda, CA USA; 4 Department of Neurosurgery and Anesthesiology, School of Medicine, Loma Linda University, Loma Linda, CA USA; 5 School of Anesthesiology, Naval Medical University, Shanghai, China; 6 Faculty of Chinese Medicine and State Key Laboratory of Quality Research in Chinese Medicines, Macau University of Science and Technology, Macau, Macau SAR China; 7 Tongji Medical College, Huazhong University of Science and Technology, Wuhan, China; 8 Guangzhou University of Chinese Medicine, Guangzhou, Guangdong China; 9 Department of Sports Medicine, Huashan Hospital, Fudan University, Shanghai, China; 10 Department of Plastic and Reconstructive Surgery, Shanghai Ninth People’s Hospital, Shanghai Jiao Tong University School of Medicine, Shanghai, China; 11 Kunming University of Science and Technology, Chenggong Campus, Kunming, People’s Republic of China; 12 Clinical Laboratory Center, The People’s Hospital of Xinjiang Uygur Autonomous Region, Ürümqi, China; 13 Department of Neurosurgery, The Affiliated Hospital, Southwest Medical University, Luzhou, Sichuan China; 14 Neurosurgical Intensive Care Unit, Department of Neurosurgery, Southwest Hospital, Third Military Medical University (Army Medical University), Chongqing, China

**Keywords:** Diseases, Medical research

## Abstract

Spontaneous intracerebral hemorrhage (ICH), especially hypertensive basal ganglia hemorrhage, is a severe, often fatal stroke subtype with high morbidity and mortality. This historical review traces therapeutic progress from the 1960s to the present, emphasizing rehabilitation, peripheral–central nervous system interactions, and clinical outcomes. Molecular mechanisms, early treatments, and advances in interventions are examined. Rehabilitation strategies, such as exercise programs, stem cell therapies, and novel physical modalities, are evaluated for their effects on neuroprotection, neuroplasticity, and functional recovery. Pharmacological approaches during subacute and chronic phases are also reviewed. Clinical trials (phases I–III) are critically assessed for end points and efficacy of combined therapies. This Review highlights emerging paradigms targeting peripheral–central pathways, personalized rehabilitation, and future directions, while addressing research limitations and clinical challenges.

**Figure:**
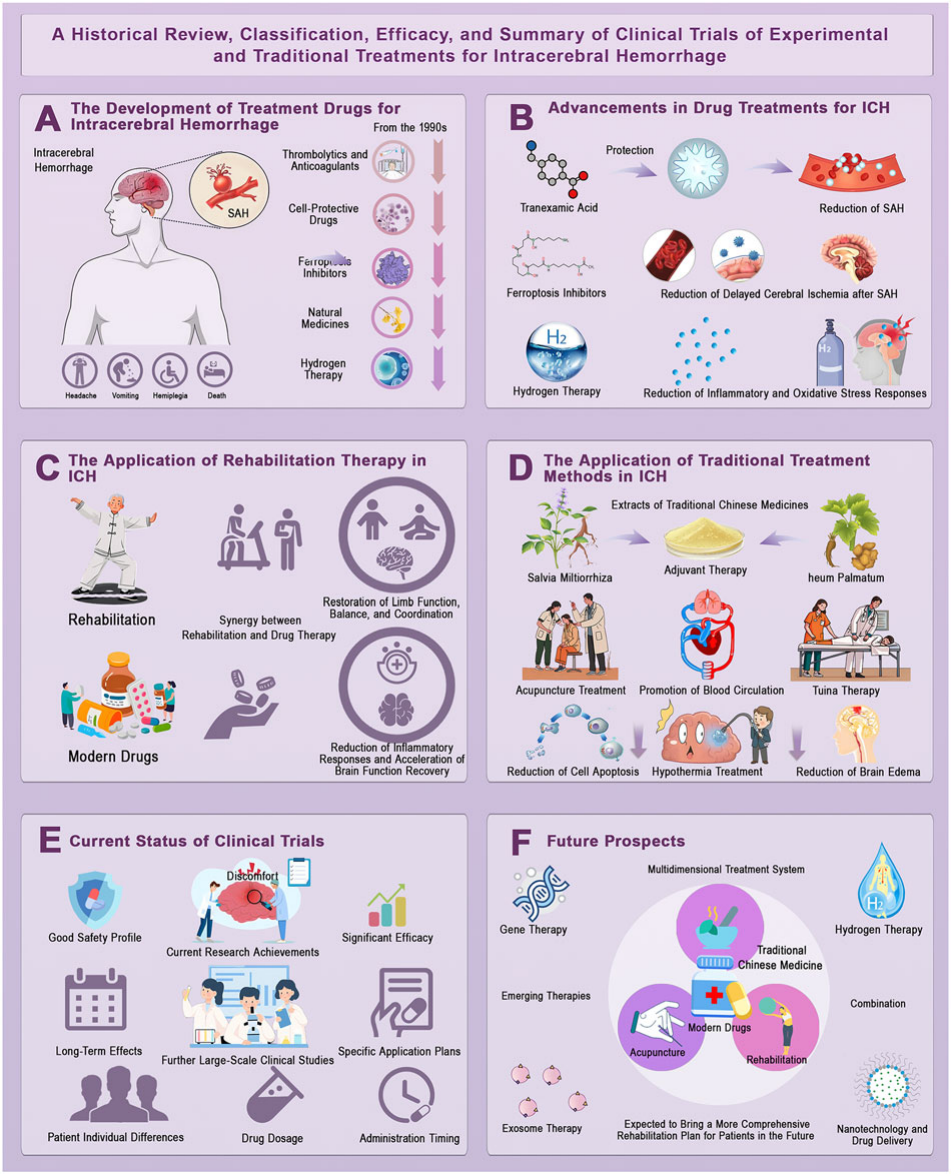
**Evolution of treatment strategies for intracerebral hemorrhage (ICH). a**, Development of treatment drugs for intracerebral hemorrhage. Since the 1990s, pharmacological interventions have evolved, including thrombolytics, anticoagulants, cell-protective drugs, ferroptosis inhibitors, natural medicines, and hydrogen therapy, all aimed at improving patient outcomes. **b**, Advancements in drug treatments for ICH. Recent therapeutic innovations focus on reducing ICH-related damage, including the use of tranexamic acid for hemorrhage control, ferroptosis inhibitors for preventing delayed cerebral ischemia, and hydrogen therapy to mitigate inflammatory and oxidative stress responses. **c**, Application of rehabilitation therapy in ICH. Rehabilitation strategies, including physical therapy and pharmacological support, promote functional recovery by enhancing limb function, balance, and coordination while reducing inflammatory responses and accelerating neuroplasticity. **d**, Traditional treatment methods for ICH. Various traditional Chinese medicine approaches, such as *Salvia miltiorrhiza*-based adjuvant therapy, acupuncture for blood circulation enhancement, Tuina therapy, and hypothermia treatment, contribute to reducing cell apoptosis and brain edema. **e**, Current status of clinical trials. Ongoing trials assess drug safety, efficacy, and application across different patient populations, whereas challenges such as patient variability, drug dosage optimization, and administration timing remain crucial for translating research into clinical practice. **f**, Future prospects. The integration of multidimensional treatment approaches, encompassing gene therapy, exosome therapy, nanotechnology-based drug delivery, hydrogen therapy, and the combination of modern pharmacology with traditional medicine, holds significant promise for refining rehabilitation protocols and enhancing patient outcomes.

**Evolution of treatment strategies for intracerebral hemorrhage (ICH). a**, Development of treatment drugs for intracerebral hemorrhage. Since the 1990s, pharmacological interventions have evolved, including thrombolytics, anticoagulants, cell-protective drugs, ferroptosis inhibitors, natural medicines, and hydrogen therapy, all aimed at improving patient outcomes. **b**, Advancements in drug treatments for ICH. Recent therapeutic innovations focus on reducing ICH-related damage, including the use of tranexamic acid for hemorrhage control, ferroptosis inhibitors for preventing delayed cerebral ischemia, and hydrogen therapy to mitigate inflammatory and oxidative stress responses. **c**, Application of rehabilitation therapy in ICH. Rehabilitation strategies, including physical therapy and pharmacological support, promote functional recovery by enhancing limb function, balance, and coordination while reducing inflammatory responses and accelerating neuroplasticity. **d**, Traditional treatment methods for ICH. Various traditional Chinese medicine approaches, such as *Salvia miltiorrhiza*-based adjuvant therapy, acupuncture for blood circulation enhancement, Tuina therapy, and hypothermia treatment, contribute to reducing cell apoptosis and brain edema. **e**, Current status of clinical trials. Ongoing trials assess drug safety, efficacy, and application across different patient populations, whereas challenges such as patient variability, drug dosage optimization, and administration timing remain crucial for translating research into clinical practice. **f**, Future prospects. The integration of multidimensional treatment approaches, encompassing gene therapy, exosome therapy, nanotechnology-based drug delivery, hydrogen therapy, and the combination of modern pharmacology with traditional medicine, holds significant promise for refining rehabilitation protocols and enhancing patient outcomes.

## Advances in treatment strategies for intracerebral hemorrhage

Spontaneous intracerebral hemorrhage (ICH) is a severe stroke type affecting millions globally, with high early mortality and long-term impairments. Recent studies highlight the shift from supportive care to mechanism-driven interventions targeting oxidative stress, inflammation, and ferroptosis, a form of cell death. Researchers are exploring pharmacological treatments such as antihypertensive therapy, neuroprotective compounds, and ferroptosis inhibitors. For instance, deferoxamine, an iron chelator, shows promise in reducing oxidative stress and neuronal damage. These findings are significant as they address the complex interplay of secondary injury mechanisms in ICH, offering potential for improved patient outcomes. The integration of pharmacological and rehabilitation strategies, including structured exercise, aims to enhance neuroplasticity and recovery. Future research should focus on precision medicine approaches, optimizing interventions based on individual patient profiles to advance ICH treatment and improve quality of life.

This summary was initially drafted using artificial intelligence, then revised and fact-checked by the author.

## Introduction

Spontaneous intracerebral hemorrhage (ICH) is one of the most devastating types of stroke, affecting nearly two million individuals worldwide each year. Despite advances in emergency medicine and neurocritical care, early mortality remains high, and many survivors continue to experience long-term impairments^1^. Unlike traumatic hemorrhage that arises from external mechanical injury, spontaneous ICH typically results from intrinsic vascular pathology such as chronic hypertension, cerebral small vessel disease, or the use of anticoagulants^1^.

The pathophysiology of ICH is commonly divided into primary and secondary brain injury. Primary injury is caused by the immediate mass effect of the hematoma, leading to compression, ischemia, and mechanical destruction of surrounding brain tissue^2^. Secondary injury evolves over hours to days and exerts a stronger impact on long-term outcomes. It involves multiple processes including the breakdown of red blood cells, release of hemoglobin and iron, disruption of the blood–brain barrier (BBB), oxidative stress, neuroinflammation, and various forms of programmed cell death^3^. Among these mechanisms, iron overload and ferroptosis, which is an iron-dependent form of cell death characterized by lipid peroxidation, have been recognized as central contributors to perihematomal edema and progressive neuronal loss. Clinically, ICH is characterized by the sudden onset of focal neurological deficits that may rapidly progress to coma or death. CT remains the gold standard for diagnosis because it enables rapid localization of the hematoma, assessment of hematoma volume, and detection of hematoma expansion^4^. MRI, particularly susceptibility-weighted imaging and ultra-high field sequences, provides additional information about microbleeds, perihematomal edema, and underlying conditions such as cerebral amyloid angiopathy^5^. Prognosis is notably influenced by hematoma volume and the extent of secondary injury processes, unlike aneurysmal subarachnoid hemorrhage, in which delayed vasospasm has a central role in determining the outcome^4,6^.

**Fig. 1:**
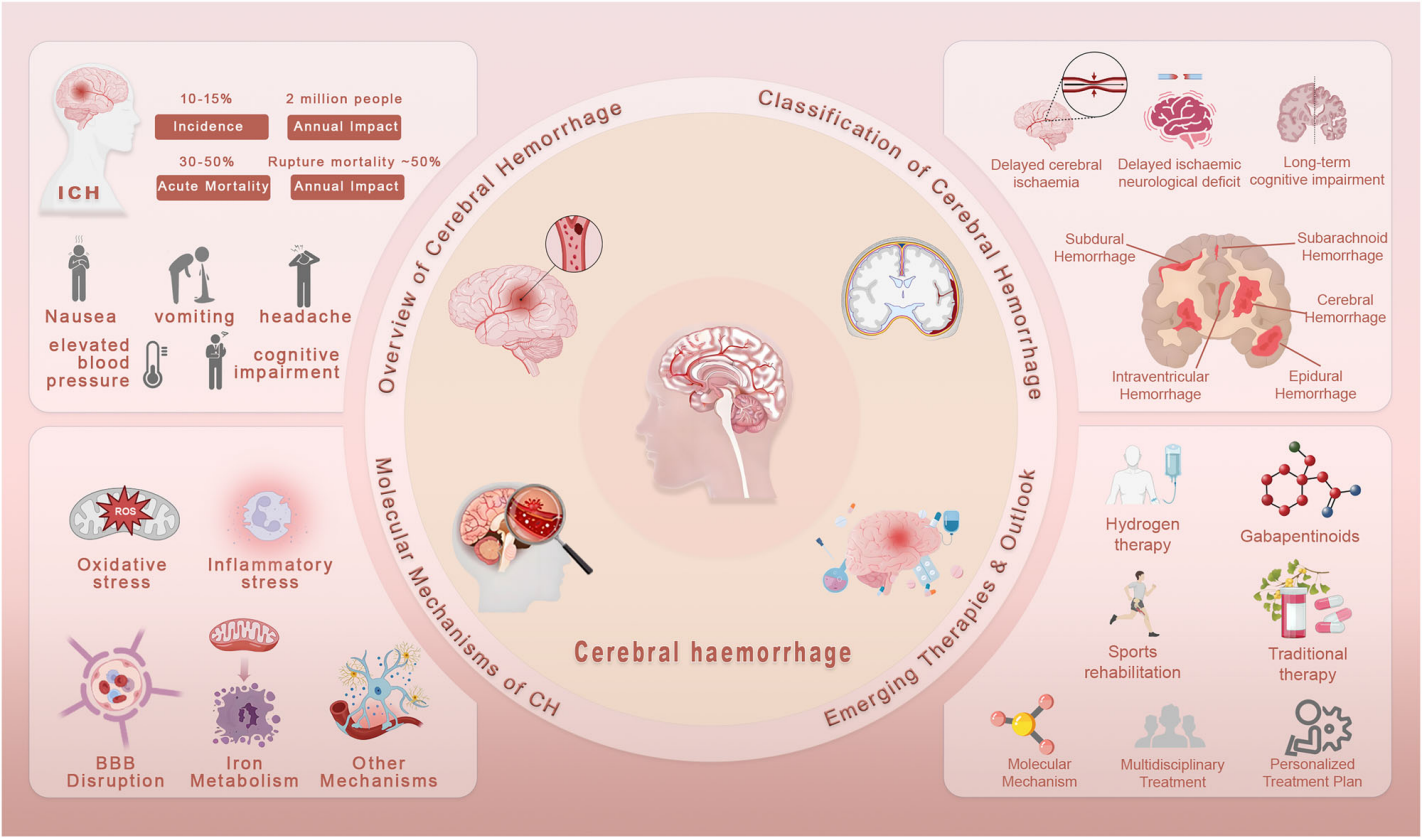
Complications, molecular mechanisms, and comprehensive treatment strategies of cerebral hemorrhage. This figure presents the categorization, complications, molecular mechanisms, and therapeutic strategies associated with cerebral hemorrhage. Hemorrhages are classified into intracerebral hemorrhage and subarachnoid hemorrhage. The section on complications emphasizes adverse outcomes that may arise from medical interventions, including delayed cerebral ischemia, delayed ischemic neurological deficit, and long-term cognitive impairment. The molecular mechanism panel illustrates the underlying pathophysiological processes, with particular attention to oxidative stress, inflammatory stress, and perihematoma edema, and highlights the nuclear factor erythroid 2-related factor 2 signaling pathway together with its regulation by Kelch-like ECH-associated protein 1. The therapy panel describes a range of treatment strategies, encompassing hydrogen therapy, gabapentinoids, sports rehabilitation, and traditional approaches, thereby underscoring the necessity of a multifaceted approach to the management of cerebral hemorrhage. BBB blood–brain barrier, ICH intracerebral hemorrhage, ROS reactive oxygen species.

In recent years, treatment strategies for ICH have gradually shifted from purely supportive care toward mechanism-driven interventions. Pharmacological approaches targeting oxidative stress, inflammation, and ferroptosis are receiving increasing attention^7^. At the same time, rehabilitation strategies, including structured exercise programs, are being recognized as essential for long-term functional recovery^8^. The integration of pharmacological and non-pharmacological therapies offers a promising pathway to enhance neuroplasticity, accelerate neurological restoration, and improve quality of life (Fig. 1). Despite this progress, the biological complexity of ICH continues to present challenges. Secondary injury reflects a dynamic interplay of mechanisms that can be both harmful and beneficial. For example, neuroinflammation may contribute to debris clearance and tissue repair when moderate, but excessive activation amplifies neuronal damage^9^. Similarly, oxidative stress and cell death pathways interact with each other in complex ways that complicate therapeutic targeting^10^. These observations highlight the need for precision medicine strategies that minimize detrimental responses while preserving protective ones.

Emerging insights into oxidative stress, iron metabolism, and cell death programs such as ferroptosis are reshaping our understanding of ICH pathophysiology and driving the search for new pharmacological treatments. Future research should focus on refining ferroptosis-targeted interventions, optimizing antioxidant strategies centered on the Nrf2–HO-1 axis, and exploring molecular and epigenetic regulators of secondary brain injury. By translating these mechanistic breakthroughs into clinical practice, the field may achieve disruptive advances in pharmacological treatment and substantially improve outcomes for patients with ICH.

## Development of pharmacological and biological therapies for intracerebral hemorrhage

### Early pharmacological attempts (1960s–1980s)

**Fig. 2:**
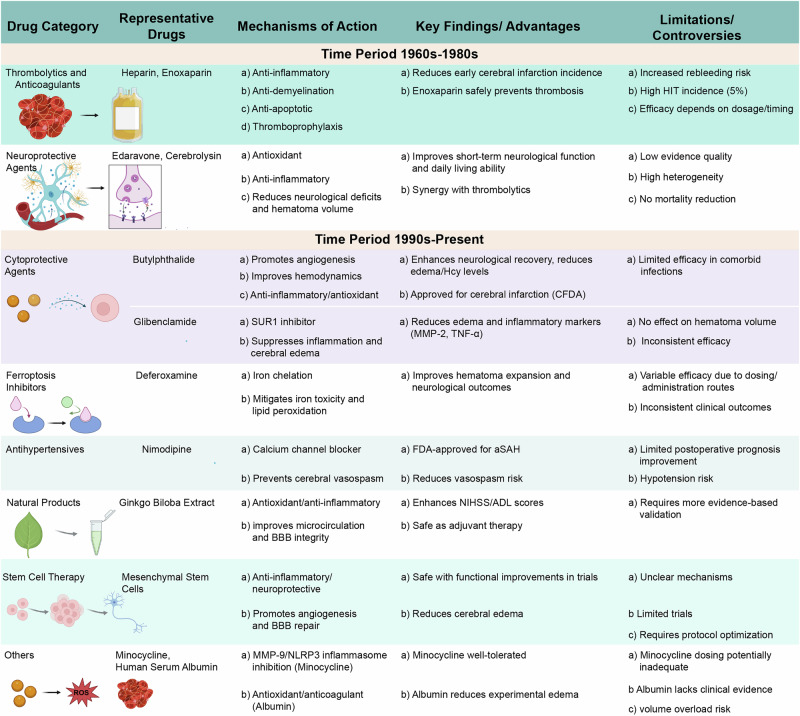
Advances in pharmacological and biological therapies for spontaneous intracerebral hemorrhage. This figure summarizes the development of therapeutic strategies for spontaneous intracerebral hemorrhage. Early efforts in the 1960s to 1980s relied on anticoagulants, thrombolytics, and first-generation neuroprotectants but showed limited efficacy and safety concerns. Since the 1990s, pharmacological approaches have focussed on mechanisms such as oxidative stress, excitotoxicity, ferroptosis, and blood–brain barrier (BBB) disruption. Representative agents include edaravone, glibenclamide, minocycline, deferoxamine, and human serum albumin, as well as natural bioactive compounds. In addition, biological therapies including mesenchymal stem cells and exosome-based interventions have emerged, offering regenerative and paracrine benefits. MMP-9 matrix metalloproteinase-9, ROS reactive oxygen species, TNF-α tumor necrosis factor-alpha, NIHSS National Institutes of Health Stroke Scale, ADL activities of daily living, NLRP3 NOD-like receptor pyrin domain-containing 3, aSAH aneurysmal subarachnoid hemorrhage, CFDA China Food and Drug Administration, Hcy homocysteine, HIT heparin-induced thrombocytopenia.

The earliest explorations into drug therapy for spontaneous ICH, particularly hypertensive basal ganglia hemorrhage, were largely empirical. Investigations during the 1960s–1980s focussed on anticoagulants, thrombolytics, and first-generation neuroprotectants. Although these strategies laid theoretical foundations, they were hampered by limited mechanistic understanding, inconsistent efficacy, and frequent adverse events. For example, heparin was tested owing to its anti-inflammatory and anti-apoptotic potential, but clinical use was compromised by heparin-induced thrombocytopenia, which was particularly pronounced in ICH populations^11^ (Fig. 2). Another candidate, edaravone, a free radical scavenger, reduced perihematomal edema and transiently improved neurological function in preclinical and early clinical settings. However, its long-term efficacy remained inconclusive, and high cost restricted its routine adoption^12^ (Fig. 2). Despite these shortcomings, early work provided key lessons on drug timing, dosing strategies, and the necessity of patient stratification. These insights set the stage for more mechanism-driven therapeutic strategies in subsequent decades^13^.

### Mechanism-oriented pharmacological advances (1990s–present)

#### Antihypertensive therapy and hemodynamic modulation

Hypertension has long been recognized as the strongest predictor of hematoma expansion and rebleeding, making antihypertensive therapy a central focus^14^. Mechanistic studies show that acute blood pressure (BP) reduction decreases shear stress at the rupture site, limits perihematomal ischemia, and reduces the likelihood of rebleeding. At the cellular level, vascular smooth muscle cells and endothelial cells respond dynamically to BP changes through ion channel regulation and mechanosensitive signaling cascades such as MAPK and RhoA/ROCK pathways^15^. Dysregulation of these processes compromises cerebral autoregulation, thereby amplifying the risk of both hematoma growth and hypoperfusion. Recent work using single-cell transcriptomics has revealed endothelial heterogeneity and patient-specific vascular tone signatures. These discoveries suggest that individualized BP management, guided by genetic polymorphisms, endothelial reactivity, or circulating biomarkers such as endothelin-1 and nitric oxide metabolites, could optimize hemodynamic stability and advance BP-lowering strategies beyond uniform protocols^16^.

#### Neuroprotective and cytoprotective compounds

Neuroprotective and cytoprotective compounds have gained prominence as potential therapies to reduce secondary brain injury, which is largely mediated by excitotoxicity, oxidative stress, apoptosis, and BBB disruption. Dl-3-*n*-butylphthalide enhances collateral circulation by promoting endothelial nitric oxide synthase activity and activates neurotrophic signaling pathways such as brain-derived neurotrophic factor/tropomyosin receptor kinase B (TrkB) and calcitonin gene-related peptide (CGRP), thereby improving synaptic plasticity and cognitive recovery^17^ (Fig. 2). Glibenclamide acts through inhibition of SUR1–TRPM4 channels, which are upregulated after ICH and drive ion influx, cellular swelling, and BBB leakage^18^. By restoring ion homeostasis and tight junction proteins such as occludin and claudin-5, glibenclamide stabilizes the neurovascular unit. Minocycline, beyond suppressing matrix metalloproteinase-9 (MMP-9), interferes with microglial activation and NLRP3 inflammasome assembly, thereby attenuating neuroinflammation^19^ (Fig. 2). It also interacts with the p53 pathway, limiting apoptosis of perihematomal neurons^20^. These agents collectively illustrate the emergence of a multitarget paradigm that simultaneously addresses vascular, glial, and neuronal injury^21^.

#### Antioxidant and anti-inflammatory strategies

Oxidative stress and neuroinflammation represent another critical therapeutic target. Heme and hemoglobin degradation products catalyze reactive oxygen species (ROS) generation, whereas damage-associated molecular patterns activate Toll-like receptors on microglia and astrocytes, driving inflammatory cascades. Tirilazad mesylate, a 21-aminosteroid, interrupts lipid peroxidation, preserves mitochondrial integrity, and improves cerebral perfusion in preclinical models^22^. Similarly, MMP inhibitors such as doxycycline and marimastat limit BBB breakdown by preserving extracellular matrix proteins and tight junctions^23^. However, the dual role of MMPs in both injury and tissue repair underscores the importance of temporal precision. Early inhibition can prevent BBB leakage, whereas delayed suppression may interfere with angiogenesis and remodeling. These observations emphasize the value of biomarker-guided therapeutic windows for antioxidant and anti-inflammatory strategies^24^.

#### Ferroptosis inhibition

The discovery of ferroptosis, an iron-dependent form of regulated cell death, has reshaped ICH research. Hemolysis after ICH releases hemoglobin and free iron, which catalyze lipid peroxidation and amplify oxidative neuronal injury^25^. DFX, a clinically available iron chelator, has shown consistent benefits in reducing lipid ROS and perihematomal edema in preclinical models, although translational outcomes have been variable owing to pharmacokinetic limitations^26^ (Fig. 2). Beyond DFX, newer compounds such as deferasirox, dauricine, and *N*-acetylcysteine offer alternative approaches by enhancing glutathione peroxidase 4 activity and replenishing antioxidant defenses^27^. Mechanistic studies also link ferroptosis inhibition with microglial polarization toward reparative M2 phenotypes and mitochondrial preservation, suggesting broader implications for both neuroprotection and immunomodulation.

#### Human serum albumin as a multifunctional neuroprotectant

Human serum albumin (HSA), traditionally viewed as an oncotic regulator, has been repurposed as a multifunctional therapeutic agent^28^. HSA binds transition metals such as iron and copper, thereby reducing ROS production through limitation of Fenton chemistry^29^. It also interacts with nitric oxide to form S-nitrosothiols, which suppress platelet aggregation and vascular inflammation. Moreover, HSA activates the ERK/Nrf2/HO-1 signaling cascade, linking it to anti-apoptotic and cytoprotective functions^30^. These diverse activities highlight pleiotropic potential of HSA, although risks such as volume overload and pulmonary edema underscore the need for optimized formulations, including nanoparticle-based or targeted delivery systems (Fig. 2).

#### Natural products and novel bioactive compounds

Natural products and novel bioactive compounds are increasingly explored for their multifaceted effects. Ginkgo biloba extract improves microcirculation, reduces pro-inflammatory cytokine release, and enhances synaptic plasticity^31^ (Fig. 2). Hydrogen therapy functions as a selective ROS scavenger, mitigating oxidative stress and apoptosis. Additional phytochemicals such as astragaloside IV, puerarin, and resveratrol modulate AMPK/Nrf2 pathways and ferroptosis-related mechanisms, often demonstrating synergistic effects with conventional neuroprotectants^32^. These compounds may serve as scaffolds for drug development and as adjuncts in multimodal regimens. In addition, several exploratory pharmacological candidates, although still at an early stage of investigation, have demonstrated preliminary potential in ICH therapy. Cilostazol, a phosphodiesterase-3 inhibitor, exerts both vasodilatory and antiplatelet effects that may improve cerebral perfusion and reduce vasospasm^33^. The mitochondria-targeted antioxidant MitoQ has shown the ability to suppress NLRP3 inflammasome activation and promote microglial M2 polarization, thereby mitigating oxidative stress and neuroinflammation^34^. Immunomodulatory approaches such as IL-27 are being studied for their capacity to regulate neutrophil polarization and inflammatory cascades^35^. Hemodynamic agents including milrinone and dobutamine are also under exploration for carefully selected patients, given their ability to enhance cerebral perfusion, although safety concerns necessitate rigorous patient stratification^36^. Collectively, these emerging candidates remain largely experimental, requiring further mechanistic studies and large-scale clinical trials to validate their efficacy and safety.

### Stem cell-based and exosome-based therapies

Beyond pharmacological agents, biological therapies such as stem cells and exosomes represent a transformative frontier in ICH management^37^. Mesenchymal stem cells and neural stem cells exert their therapeutic benefits largely through paracrine effects rather than direct neuronal replacement^38^ (Fig. 2). They have been shown to preserve BBB integrity by upregulating tight junction proteins and downregulating aquaporin-4 expression, to modulate immune balance by suppressing pro-inflammatory cytokines such as IL-6 and tumor necrosis factor-α while enhancing anti-inflammatory mediators including IL-10 and transforming growth factor-β, and to promote angiogenesis through vascular endothelial growth factor release^39^. Exosome-based therapies are emerging as a promising cell-free alternative. Exosomes derived from mesenchymal stem cells can cross the BBB and deliver trophic factors, thereby replicating many of the protective benefits of stem cells without risks such as tumorigenesis or embolization. Preclinical models consistently demonstrate neurological recovery, whereas early clinical studies report favorable safety profiles^40^. Nonetheless, challenges persist in defining optimal transplantation timing, refining delivery routes, ensuring long-term safety, and scaling up exosome production. Integration with pharmacological interventions, advanced imaging for therapeutic monitoring, and biomarker-guided treatment protocols is considered crucial for successful clinical translation.

The evolution of ICH therapy reflects a shift from empirical drug testing toward mechanism-guided, multitarget, and biologically integrated approaches. Early failures underscored the complexity of ICH, whereas contemporary strategies emphasize antihypertensive therapy, neuroprotective and cytoprotective compounds, antioxidant and anti-inflammatory agents, ferroptosis inhibitors, multifunctional proteins such as HSA, and bioactive natural products. Stem cell-based and exosome-based therapies further enrich the therapeutic arsenal by introducing biological and regenerative dimensions.

## Advances in drug therapy for spontaneous intracerebral hemorrhage

### Early research and traditional agents

**Fig. 3:**
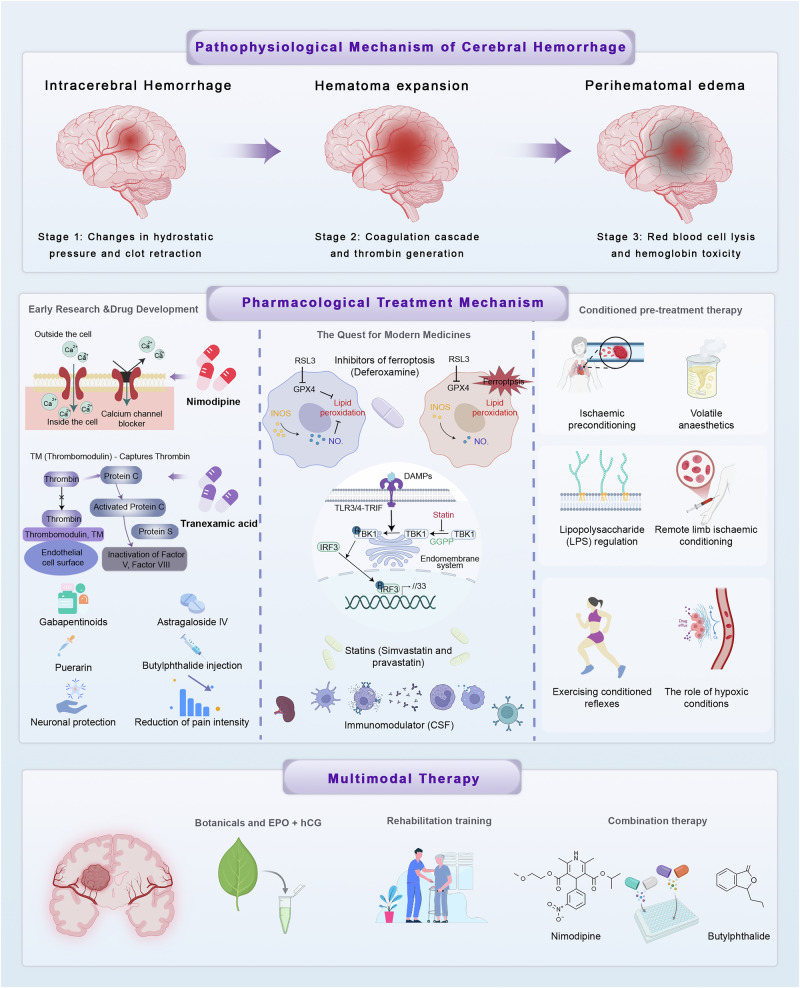
Advances in the pharmacological treatment of spontaneous cerebral hemorrhage. This image illustrates the progression and diversity of treatment strategies for spontaneous cerebral hemorrhage, highlighting early research and drug development with medications such as nimodipine, tranexamic acid, gabapentinoids, astragaloside IV, and puerarin. It also emphasizes the quest for modern medicines, including inhibitors of ferroptosis such as deferoxamine, immunomodulators, and statins. The image further details conditioned pretreatment therapies involving ischemic preconditioning, volatile anesthetics, and LPS regulation, as well as multimodal approaches such as remote limb ischemic conditioning, exercising conditioned reflexes, and the use of botanicals combined with EPO+hCG, considering the role of hypoxic conditions. Additionally, it points to endovascular therapy as a potential treatment avenue, reflecting the ongoing research and the comprehensive nature of current pharmacological and therapeutic advancements in managing cerebral hemorrhage. CSF colony-stimulating factor, DAMP damage-associated molecular pattern, EPO erythropoietin, GPX4 glutathione peroxidase 4, hCG human chorionic gonadotropin, INOS inducible nitric oxide synthase, NO, nitric oxide, TLR Toll-like receptor.

**Fig. 4:**
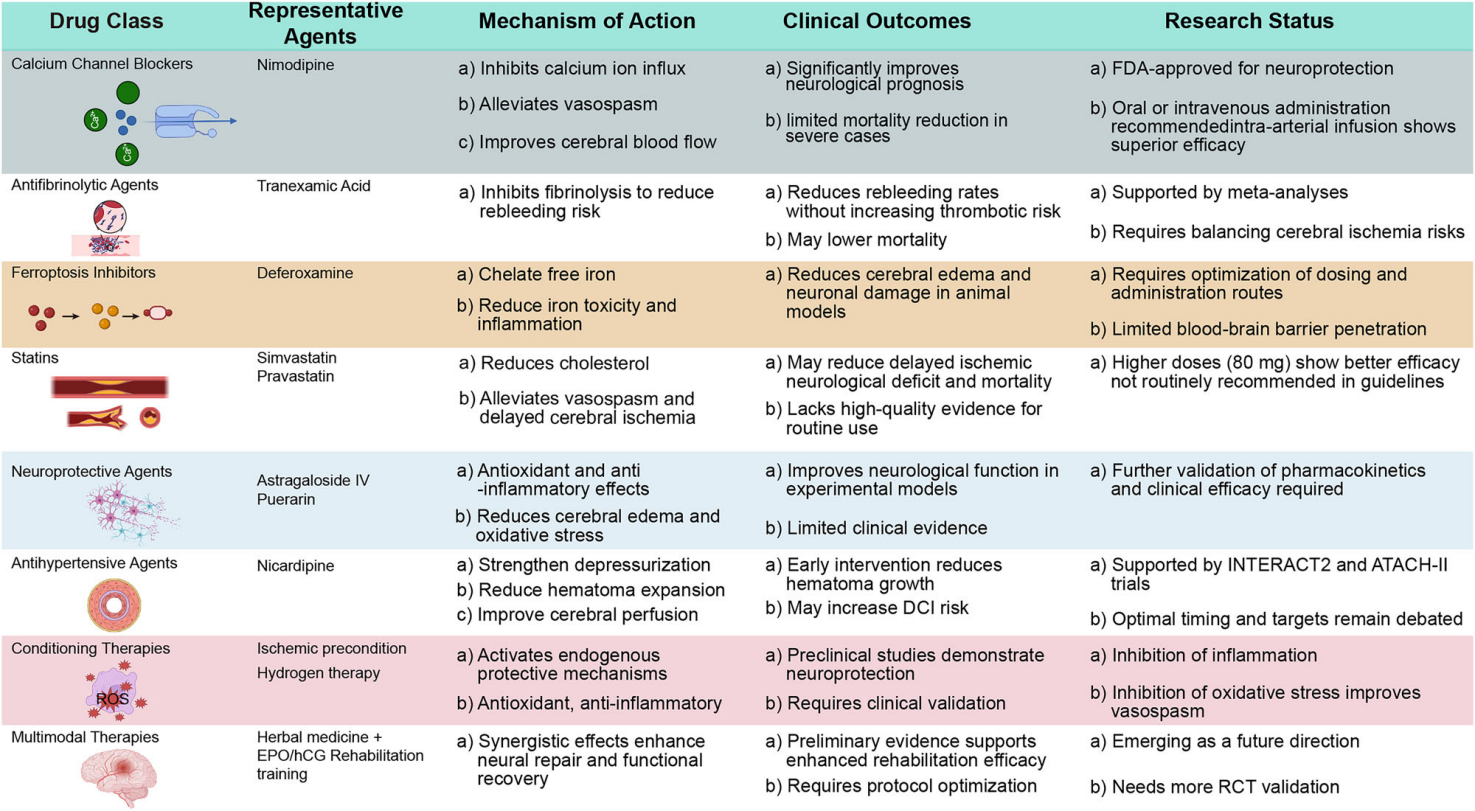
Progress in pharmacological treatment of spontaneous intracerebral hemorrhage. This figure summarizes advances in pharmacological strategies for spontaneous intracerebral hemorrhage. Calcium channel blockers such as nimodipine improve cerebral perfusion and alleviate vasospasm but provide limited survival benefit. Antifibrinolytic agents including tranexamic acid reduce rebleeding risk without consistently improving long-term outcomes. Ferroptosis inhibitors such as deferoxamine mitigate iron toxicity and oxidative stress yet face challenges with dosing and delivery. Statins show pleiotropic vascular and anti-inflammatory effects but remain controversial in clinical practice. Natural compounds such as astragaloside IV and puerarin demonstrate antioxidant and neuroprotective actions with limited clinical validation. Conditional preconditioning strategies and multimodal approaches that combine pharmacological agents with rehabilitation represent emerging directions for integrated intracerebral hemorrhage management. DCI decompression illness, EPO erythropoietin, hCG human chorionic gonadotropin, RCT randomized controlled trial, ROS reactive oxygen species.

Pharmacological interventions for spontaneous ICH initially centered on drugs that were already in use for other cerebrovascular conditions. These early attempts were pragmatic, guided more by availability and pathophysiological assumptions than by dedicated mechanistic evidence. Nimodipine, a calcium channel antagonist successfully applied in aneurysmal subarachnoid hemorrhage, was among the first tested in ICH (Fig. 3). The rationale was that by improving cerebral perfusion and alleviating vasospasm, nimodipine might attenuate ischemia in perihematomal regions. Clinical studies revealed modest improvements in neurological function in selected subgroups, particularly when given early, yet survival benefits in patients who are severely ill were inconsistent^41^ (Fig. 4). These findings highlighted that cerebral vasospasm, a key driver of outcome in subarachnoid hemorrhage, has a less dominant role in ICH, thereby limiting the translatability of benefits of nimodipine. Tranexamic acid, an antifibrinolytic agent, was introduced to reduce hematoma expansion by inhibiting clot breakdown (Fig. 3). Several meta-analyses have confirmed reductions in short-term rebleeding and hematoma growth^42^. However, tranexamic acid did not consistently improve long-term survival or functional recovery, and concerns about thromboembolic complications constrained enthusiasm (Fig. 4). This underscored the principle that reducing hematoma expansion alone may be insufficient without addressing secondary injury cascades. Dl-3-*n*-butylphthalide, originally approved for ischemic stroke in China, has also been tested in ICH (Fig. 3). Small-scale studies suggested enhanced cerebral perfusion, reduced oxidative stress, and modest improvements in functional outcomes, especially when used with nimodipine^43^. Nevertheless, high-quality randomized controlled trials (RCTs) remain lacking. In parallel, natural compounds such as astragaloside IV and puerarin, and adjunctive gabapentinoids for neuropathic pain, have been used with variable success^44^ (Fig. 3). Collectively, these early explorations revealed the potential but also the limitations of simply repurposing existing drugs, emphasizing the need for therapies tailored to the unique biology of ICH (Fig. 4).

### Modern drug explorations

#### Antihypertensive therapy

Elevated BP after ICH onset is one of the most important modifiable predictors of poor outcome, strongly associated with hematoma expansion, perihematomal edema, and early neurological deterioration^45^. Consequently, antihypertensive therapy has remained the backbone of acute ICH management (Fig. 4). Large-scale RCTs have provided valuable but divergent insights. The INTERACT2 trial demonstrated that intensive BP lowering was associated with improved functional outcomes, although absolute risk reduction was modest. By contrast, the ATACH-II trial, which applied more aggressive BP reduction, failed to demonstrate significant benefits and raised concerns about renal adverse events^46^. These conflicting findings illustrate the narrow therapeutic window between preventing hematoma expansion and inducing cerebral hypoperfusion.

Mechanistically, acute BP reduction reduces shear stress on fragile vessels and limits secondary bleeding. At the vascular level, endothelial cells and vascular smooth muscle cells dynamically respond to BP fluctuations via ion channel activity, mechanosensitive receptors, and downstream pathways such as MAPK and RhoA/ROCK. Dysregulation of these mechanisms compromises autoregulation, potentially transforming protective therapy into secondary ischemic insult.

In clinical practice, current guidelines recommend individualized targets, typically focussing on systolic BP while avoiding sudden drops^47^. Novel strategies under investigation include biomarker-guided therapy using circulating nitric oxide metabolites or endothelin-1 levels, and genetic profiling to identify responders to specific antihypertensive agents^48^. Future directions emphasize precision titration and integration with neuromonitoring to maximize safety and efficacy.

#### Ferroptosis inhibitors

The discovery of ferroptosis, an iron-dependent form of regulated cell death, represents one of the most significant mechanistic breakthroughs in ICH research (Fig. 3). After hematoma formation, hemolysis releases hemoglobin, iron, and heme, which catalyze lipid peroxidation and generate toxic ROS. This drives neuronal death, perihematomal edema, and prolonged neuroinflammation^49^. Deferoxamine (DFX), the prototypical iron chelator, has been extensively studied. Preclinical studies consistently demonstrated reductions in hematoma size, oxidative stress, and neurological deficits. However, clinical trials have been less conclusive, often showing modest or no improvement in long-term disability indices such as the modified Rankin Scale. Variability in hematoma location, patient selection, dosing regimen, and administration routes likely account for these discrepancies (Fig. 4). Importantly, DFX exhibits a favorable safety profile, which supports its continued exploration^25^. Newer compounds, including deferasirox, dauricine, and *N*-acetylcysteine, act by enhancing glutathione peroxidase 4 activity and replenishing antioxidant defenses. Mechanistic work also suggests that ferroptosis inhibitors may shift microglial phenotypes toward reparative states and protect mitochondria, thereby offering broader neuroprotective and immunomodulatory effects. Future research will likely focus on combination regimens that integrate ferroptosis inhibition with anti-inflammatory or neuroregenerative therapies.

#### Statins

Statins, widely prescribed for dyslipidemia, have attracted attention owing to their pleiotropic effects beyond lipid lowering (Fig. 3). They improve endothelial function, attenuate oxidative stress, suppress inflammatory cytokines, and reduce vasospasm. Observational studies and meta-analyses suggest that statins may improve neurological outcomes and even reduce mortality in ICH, particularly at higher doses^50^. However, clinical evidence remains heterogeneous, with some trials showing no benefit and others raising concerns about potential increased bleeding risk. Safety in the ICH population is not fully established, and current international guidelines do not endorse statins as standard therapy for ICH^51^ (Fig. 4). Despite these limitations, the biological rationale remains compelling, and ongoing research seeks to clarify the role of statins, especially in selected subgroups such as patients with concomitant atherosclerotic disease.

#### Immunomodulators

Secondary brain injury in ICH is strongly shaped by the immune response. Microglia, macrophages, and T cells display dynamic and heterogeneous activation states, with the balance between pro-inflammatory and reparative phenotypes influencing outcome^52^. Erythropoietin has been tested for its neuroprotective and pro-angiogenic effects, but results have been inconsistent, with some trials reporting functional gains and others raising concerns about thromboembolic risks and increased mortality. Granulocyte colony-stimulating factor has shown the ability to mobilize progenitor cells and modulate immune tone, but clinical data remain inconclusive^53^. Beyond these hematopoietic agents, more targeted approaches aim to modulate cytokine cascades and immune checkpoints (Fig. 4). For example, IL-27 and small molecules that alter macrophage polarization are under investigation. Importantly, immunomodulation is unlikely to succeed as a stand-alone therapy; rather, it may prove effective when integrated with rehabilitation, as physical activity itself promotes anti-inflammatory signaling and supports neuroplasticity^54^.

#### Drugs with limited or inconsistent efficacy

Numerous pharmacological agents have shown limited or inconsistent benefits in ICH. Stimulants such as amphetamine and amantadine, and nootropics such as citicoline and piracetam, have failed to produce robust improvements in large trials^55^. These disappointments reflect the limitations of single-target approaches in a disorder characterized by multifactorial injury cascades. Nevertheless, exploration continues. Celecoxib, a selective COX-2 inhibitor, has demonstrated early potential in reducing edema and hematoma volume in multicenter studies^56^. Magnesium sulfate, once considered promising owing to its vasodilatory and neuroprotective properties, produced negative results in large phase III trials but remains under selective re-investigation. Endothelin-1 receptor antagonists such as clazosentan initially yielded poor efficacy, yet ongoing studies are refining dosing and patient selection criteria. Together, these experiences reinforce the necessity of biomarker-driven and stratified trial designs that align therapeutic mechanisms with patient-specific biological profiles.

### Conditional preconditioning therapies

Beyond conventional drugs, conditional preconditioning strategies have emerged as adjunctive interventions that exploit endogenous protective pathways^57^ (Fig. 4). Ischemic preconditioning, induced by transient sublethal ischemia, activates antioxidative and anti-inflammatory pathways, reduces apoptosis, and supports angiogenesis (Fig. 3). Key molecular mediators include endothelial nitric oxide synthase, hypoxia-inducible factor-1α, and sirtuin-1, which collectively stabilize the BBB and improve microcirculation^58^.

Pharmacological preconditioning with volatile anesthetics such as isoflurane and sevoflurane has shown neuroprotective efficacy in preclinical ICH models (Fig. 3). Sevoflurane, in particular, reduces BBB disruption, perihematomal edema, and neuronal apoptosis in a dose-dependent fashion^59^. Remote ischemic conditioning, achieved through cyclic limb ischemia and reperfusion, triggers systemic protective responses, including activation of autophagy markers such as Beclin-1 and LC3 (Fig. 3).

Exercise-based preconditioning represents a clinically feasible form of conditioning. By activating Nrf2–HO-1 antioxidative signaling, enhancing neuroplasticity, and suppressing inflammatory cytokines, structured physical activity primes the brain for resilience against secondary injury^60^ (Fig. 3). Collectively, conditional preconditioning approaches are unlikely to replace pharmacotherapy but may enhance therapeutic efficacy when integrated into multimodal regimens.

### Multimodal and rehabilitation-based therapies

The multifactorial nature of ICH underscores the limitations of single-target pharmacological strategies. Secondary brain injury involves complex and interdependent processes, including oxidative stress, inflammation, excitotoxicity, ferroptosis, and neurovascular unit disruption. Consequently, recent therapeutic paradigms increasingly advocate for multimodal approaches that integrate pharmacological agents with structured rehabilitation strategies to maximize recovery potential^61^ (Fig. 3).

The rationale for such integration lies in their complementary mechanisms of action. Pharmacological therapies, including antioxidants, neurotrophic agents, and metabolic modulators, can create a favorable biological environment by reducing oxidative damage, limiting neuroinflammation, stabilizing the BBB, and supporting angiogenesis. Exercise rehabilitation, in turn, capitalizes on this optimized microenvironment by stimulating neural plasticity, promoting synaptic remodeling, and enhancing cortical reorganization. Together, these interventions establish a synergistic framework in which drugs mitigate secondary injury while rehabilitation drives functional restoration. Clinical evidence supports this concept. Early initiation of rehabilitation following hemodynamic stabilization has been associated with reduced disability and improved independence in activities of daily living. During the acute phase, rehabilitation often involves passive or assisted exercises that prevent disuse atrophy while engaging residual neural circuits. As patients stabilize, therapy transitions toward active, task-specific programs including gait training, balance improvement, and fine motor exercises tailored to functional deficits. In the chronic stage, long-term adherence to individualized rehabilitation protocols has been shown to slow functional decline, preserve cognitive function, and improve overall quality of life. Importantly, rehabilitation is not only a physical intervention but also a driver of neurobiological recovery, as it enhances brain-derived neurotrophic factor signaling, promotes angiogenesis, and modulates immune tone toward anti-inflammatory states^62^.

The synergistic effects of combined therapy have been observed in several clinical and translational studies (Fig. 3). For example, antioxidant therapies that reduce ROS burden have been shown to amplify the plasticity-inducing effects of exercise. Similarly, drugs targeting ferroptosis or promoting angiogenesis appear to facilitate the structural and metabolic remodeling induced by physical training. Conversely, rehabilitation enhances drug responsiveness by promoting drug penetration into affected brain regions via improved cerebral perfusion and vascular remodeling. These bidirectional interactions highlight the interdependence between pharmacological and non-pharmacological strategies. Despite encouraging findings, challenges remain. Not all patients are suitable candidates for intensive combined regimens. Factors such as advanced age, comorbidities, hemodynamic instability, and poor baseline functional status may limit tolerance. Moreover, patient adherence is a critical determinant of success, requiring sustained motivation, family support, and multidisciplinary guidance. To optimize outcomes, comprehensive assessments incorporating clinical condition, neuroimaging markers, and molecular biomarkers should guide the selection and sequencing of multimodal strategies.

Looking ahead, the development of biomarker-guided multimodal therapies is a promising direction. Dynamic monitoring of inflammatory cytokines, oxidative stress markers, or ferroptosis-related molecules could inform personalized treatment plans that synchronize pharmacological and rehabilitative interventions. Integration of advanced technologies such as robotics, brain–computer interfaces, and virtual reality into exercise rehabilitation may further enhance neuroplasticity and functional gains. In parallel, combining pharmacological interventions with stem-cell-based or exosome-based therapies could provide additional regenerative support, reinforcing the benefits of rehabilitation-driven reorganization. In summary, multimodal therapy that integrates drug interventions with structured rehabilitation represents a paradigm shift in ICH management. Rather than addressing isolated injury pathways, it leverages complementary biological and functional mechanisms to promote holistic recovery. This combined approach, grounded in mechanistic insights and refined by individualized protocols, holds great potential to transform long-term outcomes for patients with ICH.

## Complementary and traditional therapies

### Traditional Chinese medicine (TCM)

Complementary therapies have long served as valuable adjuncts in the treatment of spontaneous ICH, providing multitarget actions that complement modern pharmacological strategies. Among these, TCM, including herbal medicine and acupuncture, has attracted particular attention. Recent advances in network pharmacology and high-throughput screening have revealed that bioactive compounds derived from TCM can modulate key molecular pathways, offering both mechanistic insights and a framework for the modernization of traditional medicine.

**Fig. 5:**
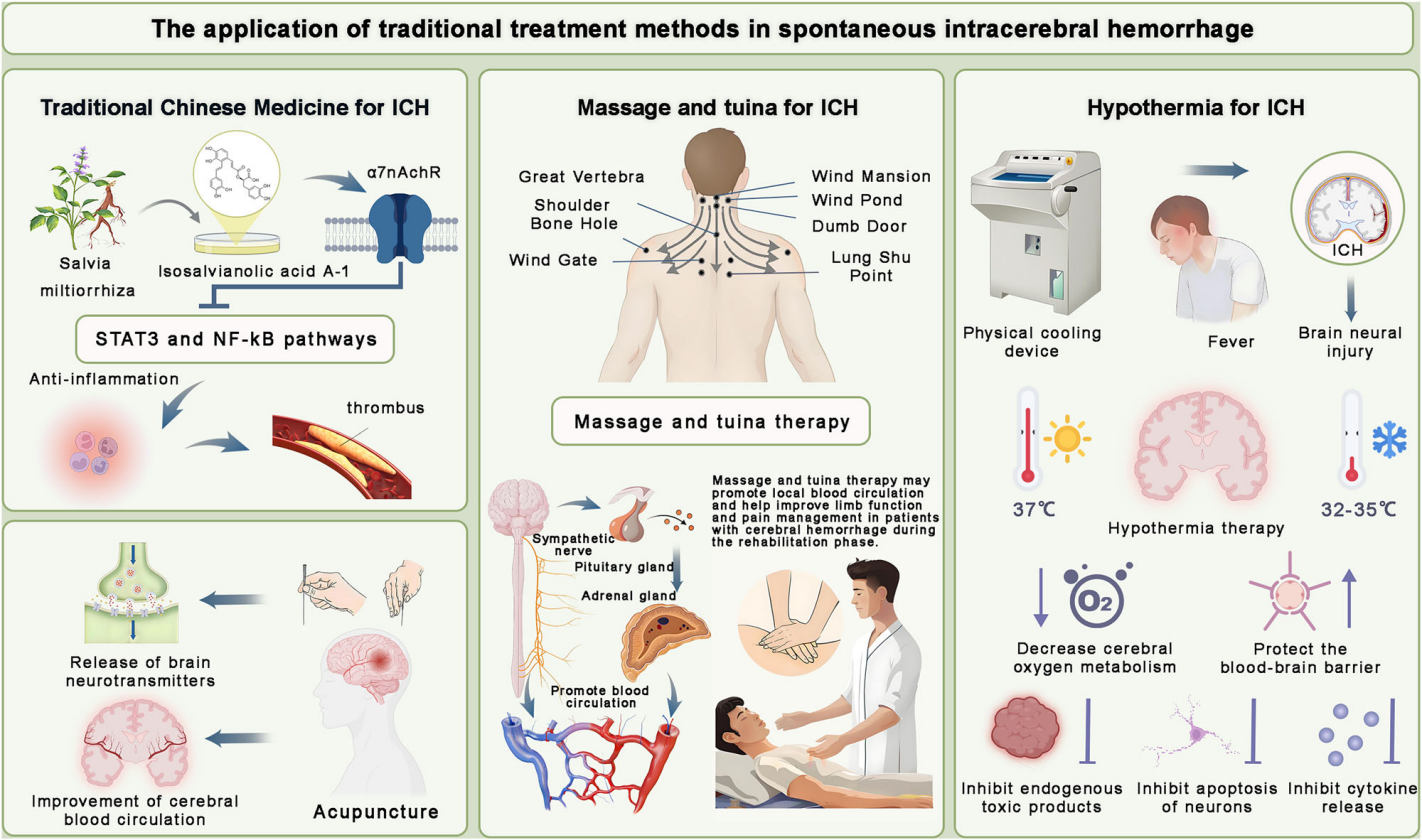
Traditional therapeutic approaches in spontaneous intracerebral hemorrhage. This figure illustrates complementary and traditional therapeutic strategies in spontaneous intracerebral hemorrhage (ICH). Herbal medicine such as *S. miltiorrhiza* and *Rheum officinale* exerts antioxidant, anti-inflammatory, anti-apoptotic, and antithrombotic effects, supporting blood–brain barrier integrity and functional recovery. Acupuncture promotes neurotransmitter release, enhances cerebral metabolism, improves microcirculation, and facilitates neuronal repair. Massage and tuina therapy improve circulation, reduce spasticity, regulate neurotransmitter balance, and promote neuroplasticity. Hypothermia therapy reduces cerebral metabolic demand, decreases edema and apoptosis, suppresses pro-inflammatory markers, and protects the blood–brain barrier. Together, these approaches highlight the multitarget benefits of traditional therapies in ICH management.

Chinese herbal medicine demonstrates multidimensional effects. *Salvia miltiorrhiza* (Danshen) contains salvianolic acids and tanshinones that exert antioxidative, anti-inflammatory, and anti-apoptotic actions. Preclinical studies suggest that Danshen extracts help maintain BBB integrity, enhance neuronal survival, and improve functional recovery^63^ (Fig. 5). *Rheum officinale* (Dahuang), traditionally used for detoxification, reduces neuroinflammation and apoptosis while facilitating neurogenesis and tissue repair^64^. Salvianolic acid injection, derived from Danshen, has shown neuroprotective effects in both animal models and clinical pilot studies, reinforcing its translational potential. Other herbs such as Panax notoginseng (Sanqi) and aesculus saponin sodium contribute to hemodynamic stabilization, microcirculatory enhancement, and long-term rehabilitation^65^.

Acupuncture represents another core modality within TCM. Mechanistic studies indicate that acupuncture regulates neurotransmitter release, improves neuronal energy metabolism, and enhances cerebral microcirculation. Its actions extend to the reduction of oxidative stress, suppression of inflammatory responses, and promotion of neuronal repair^66^ (Fig. 5). Functional imaging studies further suggest that acupuncture may facilitate cortical network reorganization by modulating activity within motor and cognitive circuits. Despite encouraging results, large-scale RCTs are still necessary to standardize acupoint selection, stimulation parameters, and treatment frequency and to validate reproducible clinical efficacy^67^.

### Massage and manual therapy

Massage and manual therapies, often applied in conjunction with acupuncture, aim to improve local circulation, reduce muscle spasticity, and enhance neuromuscular responsiveness during ICH rehabilitation. At the mechanistic level, these interventions stimulate proprioceptive pathways, promote neuroplasticity, and regulate neurotransmitter balance, which collectively contribute to functional recovery^68^ (Fig. 5). Clinical studies suggest that massage, when integrated into comprehensive rehabilitation programs, can reduce motor dysfunction and spasticity while alleviating secondary complications such as pain and joint stiffness (Fig. 5). The antioxidative and anti-inflammatory benefits observed in preclinical work also support its role in mitigating secondary injury cascades. Nevertheless, current practice is limited by heterogeneous protocols, differences in practitioner expertise, and lack of standardized outcome measures. Future directions should focus on developing evidence-based guidelines that define individualized treatment strategies and facilitate integration with modern rehabilitation frameworks^69^.

### Hypothermia therapy

Hypothermia therapy has been investigated as a non-pharmacological neuroprotective approach for ICH. By lowering cerebral metabolic demand, suppressing inflammatory activation, and preserving BBB integrity, hypothermia may attenuate secondary injury^70^ (Fig. 5). Preclinical studies consistently demonstrate reductions in edema, apoptosis, and MMP-mediated BBB disruption^71^. Clinical trials have further reported decreases in inflammatory biomarkers and improvements in cerebral perfusion^72^. Despite these promising effects, several challenges hinder clinical translation. Protocol heterogeneity, including variation in cooling depth, duration, and rewarming strategies, complicates comparison across studies. Moreover, risks of hematoma expansion, infection, and shivering-induced stress remain important safety concerns^73^. Emerging refinements such as localized cooling devices and stepwise temperature modulation have been proposed to mitigate risks and improve tolerability. With further validation in large-scale randomized trials, hypothermia may evolve into a safe and effective adjunct within comprehensive ICH management^74^ (Fig. 5).

## Drugs in clinical trials and their outcomes

### Overview of phase I–III clinical trials

In recent decades, numerous pharmacological agents have progressed into phase I–III clinical trials for spontaneous ICH. Despite promising preclinical data, clinical outcomes remain inconsistent, largely due to patient heterogeneity, complexity of trial design, and variation in dosing regimens. These challenges underscore the translational gap that persists between laboratory discoveries and bedside application.

DFX, a prototype iron chelator, has been extensively tested, given the central role of iron overload in ICH pathology. Preclinical studies demonstrated reductions in oxidative stress, neuroinflammation, and perihematomal edema, with improvements in functional recovery^75^. Phase II and phase III trials confirmed its feasibility and partial efficacy in reducing secondary brain injury; however, long-term outcomes such as survival and disability reduction remain inconclusive. Pharmacokinetic limitations and variability in treatment protocols are considered major barriers, highlighting the need for optimized delivery strategies.

**Fig. 6:**
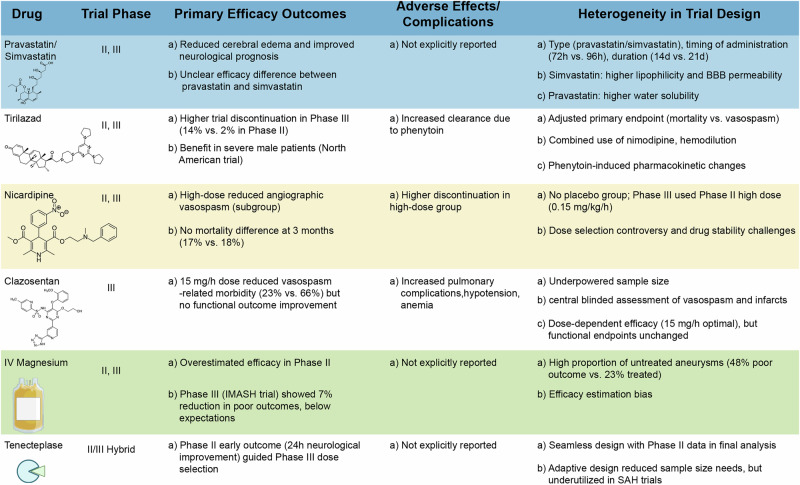
Pharmacological agents in phase II and III clinical trials for spontaneous intracerebral hemorrhage. This figure summarizes the outcomes of major drugs that have entered phase II and III clinical trials for spontaneous intracerebral hemorrhage. Agents include statins, tirilazad, nicardipine, clazosentan, intravenous magnesium, and tenecteplase. The table highlights their primary efficacy outcomes, adverse events, and sources of heterogeneity in trial design. Although some agents demonstrated biological plausibility and partial efficacy in early trials, large-scale randomized studies often produced inconsistent or negative results owing to factors such as underpowered sample sizes, patient heterogeneity, dosing regimens, and methodological limitations. These findings emphasize the challenges of translating preclinical promise into reliable clinical benefit. BBB blood–brain barrier.

Statins, commonly used for lipid-lowering therapy, have been evaluated for their pleiotropic effects including endothelial protection, vasodilation, and anti-inflammatory activity. Several observational studies suggested improved neurological recovery and reduced mortality; however, RCTs produced mixed results owing to differences in initiation timing, dosage, and treatment duration (Fig. 6). Consequently, statins are not currently recommended as standard therapy, although they remain under active investigation as potential adjuvant agents.

Among neuroprotective drugs, tirilazad, a lipid peroxidation inhibitor, showed dose-dependent benefits in early-phase trials, with stronger efficacy observed in male patients with high-grade disease. Outcomes in female patients, however, were confounded by drug–drug interactions with phenytoin^49,76^ (Fig. 6). Clazosentan, an endothelin receptor antagonist, demonstrated reductions in vasospasm-related complications and mortality at higher doses; however, its clinical application was limited by adverse effects including pulmonary complications and anemia^77^ (Fig. 6).

Magnesium sulfate, once regarded as a promising neuroprotective agent, initially demonstrated potential in reducing vasospasm during phase II trials (Fig. 6), but overestimation of its effectiveness and methodological limitations ultimately diminished its clinical appeal^78,79^. Collectively, these results reveal that agents including DFX, statins, tirilazad, clazosentan, and magnesium sulfate have yet to demonstrate consistent long-term efficacy. Discrepancies across studies reflect the complexity of ICH pathology and highlight the necessity of patient stratification, optimized dosing, standardized end points, and innovative trial designs. Emerging clinical trial methodologies, such as adaptive designs that allow protocol modification based on interim analyses and seamless phase II/III designs that integrate early data into confirmatory stages, may improve trial efficiency and accelerate translation into effective therapies^80^ (Fig. 6).

### Hydrogen therapy in clinical development

Hydrogen therapy has recently emerged as a novel neuroprotective approach owing to its favorable safety profile and multitarget actions. Preclinical studies consistently demonstrate its ability to reduce oxidative stress, modulate inflammatory cascades, and protect BBB integrity^81^. These effects are complemented by reductions in neuronal apoptosis and perihematomal edema, providing a strong mechanistic rationale for clinical translation.

Phase I clinical trials have confirmed the safety of hydrogen administration across multiple delivery routes, with no severe adverse events reported^82^. Phase II trials further demonstrated promising efficacy, including attenuation of cerebral edema, downregulation of pro-inflammatory markers, and improvements in early neurological recovery^83^. These findings suggest that hydrogen therapy may serve as both an acute-phase intervention to reduce secondary injury and a longer-term strategy to support neurovascular repair.

Despite these encouraging results, several challenges remain. Interpatient variability, optimal dosing parameters, delivery methods (inhalation, hydrogen-rich saline, or hydrogen-producing biomaterials), and therapeutic time windows require further clarification. Large-scale RCTs are ongoing to validate its long-term efficacy, impact on functional outcomes, and prognostic benefits^84^. If confirmed, hydrogen therapy may become an innovative adjunctive modality, complementing pharmacological treatments and rehabilitation programs within a multimodal management framework^85^.

Clinical trial outcomes for pharmacological agents in ICH highlight both progress and persistent challenges. Although traditional agents such as DFX and statins show mechanistic promise, inconsistent trial results emphasize the importance of precision medicine approaches, standardized protocols, and patient-specific strategies. Hydrogen therapy stands out as a novel intervention with robust preclinical support and early clinical feasibility, but requires further validation.

## Future directions and current limitations

### Emerging therapeutic directions

**Fig. 7:**
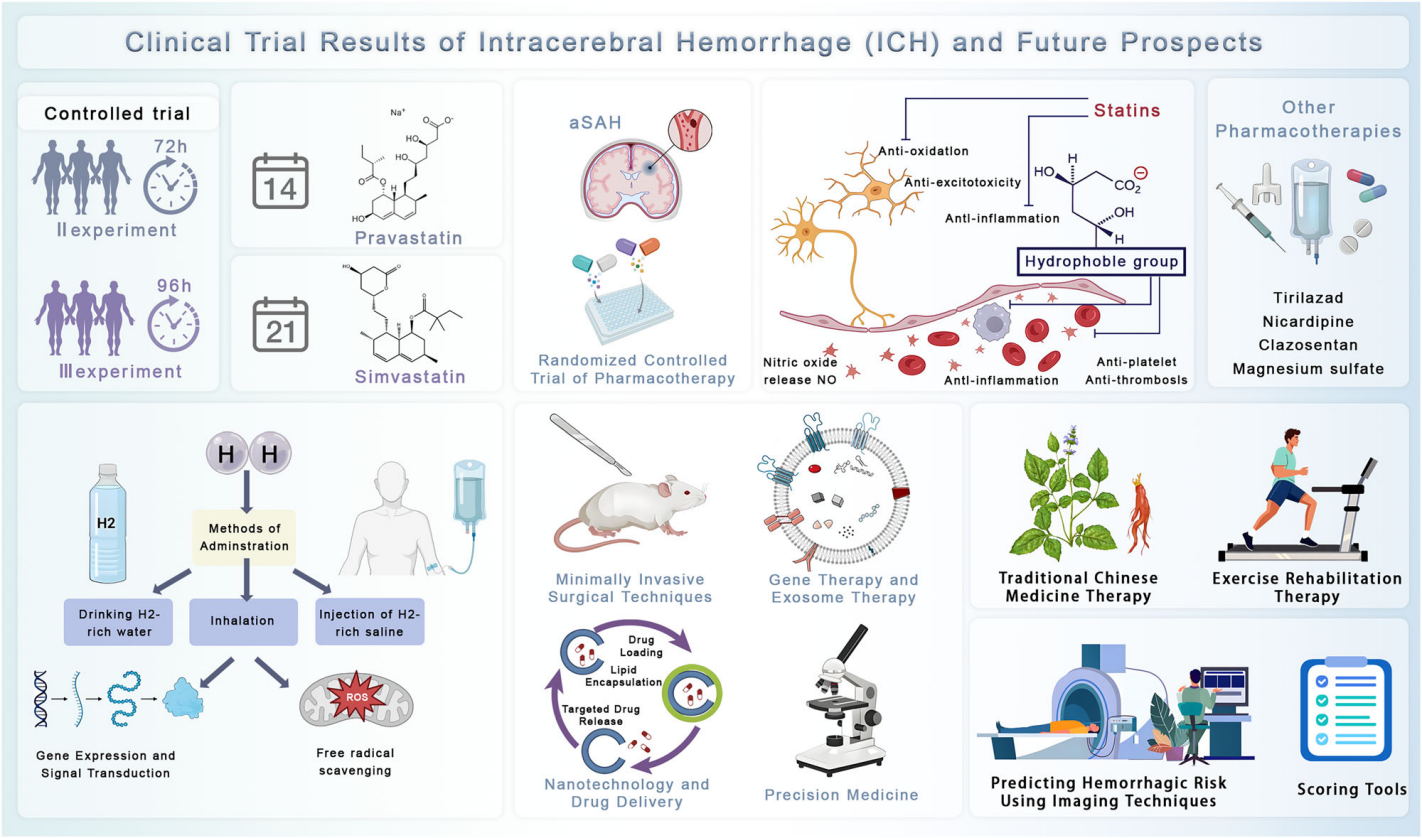
Advancements in cerebral hemorrhage treatment and research. The image outlines cutting-edge directions in cerebral hemorrhage management, featuring clinical trial drugs such as desferrioxamine and statins, minimally invasive ICH surgery, gene and nanotechnology-based therapies, and hydrogen therapy. It emphasizes precision medicine, personalized bleeding risk prediction, and ASPECTS rating for prognosis. Traditional therapies, sports rehabilitation, and advanced imaging techniques such as ECASS, T2 MRI, and dual energy CT are also highlighted, alongside the ongoing development and limitations of preclinical and clinical research. ROS reactive oxygen species.

Future strategies for ICH are increasingly shaped by the integration of advanced technologies, novel biological therapies, and precision medicine principles (Fig. 7). Minimally invasive surgery has emerged as a research priority, aiming to reduce surgical trauma, expedite hematoma clearance, and preserve functional tissue. Imaging-assisted minimally invasive surgery approaches, including navigation-guided craniopuncture, have already been standardized in some regions such as China, where they have demonstrated reduced rebleeding risk and improved outcomes^86^.

Beyond surgical innovation, biological therapies are gaining momentum. Gene therapy, exosome-based interventions, and hydrogen therapy represent promising avenues, targeting both acute neuroprotection and long-term tissue repair^87^ (Fig. 7). Advances in mitochondrial biology have introduced techniques for mitochondrial repair and intercellular transfer, offering opportunities to restore neuronal metabolism and resilience, although ethical and technical barriers remain substantial. Similarly, biomimetic materials and extracellular matrix scaffolds are under development to provide supportive microenvironments that enhance stem cell survival and differentiation^88^.

Nanotechnology is another frontier in ICH therapy. Nanocarriers enhance drug pharmacokinetics, reduce systemic toxicity, and enable precise BBB penetration^89^ (Fig. 7). Mesoporous silica nanoparticles, for example, have been engineered to deliver deferoxamine with controlled release, thereby mitigating iron-mediated oxidative injury more effectively than free drug^90^. Parallel advances in clinical trial design, such as Bayesian and adaptive models, are also expected to improve patient stratification, accelerate recruitment, and optimize dosing regimens, ultimately increasing the efficiency of drug development pipelines^91^.

### Traditional and integrative approaches

Despite rapid biomedical innovation, traditional therapies remain an important complement to modern medicine (Fig. 7). Compounds from Chinese herbal medicine, such as extracts of *S. miltiorrhiza* and baicalin, have demonstrated antioxidative, anti-inflammatory, and antithrombotic activities^92^. Acupuncture continues to show potential in modulating neurotransmitter release, improving cerebral metabolism, and promoting neural recovery^93^.

Exercise rehabilitation, bridging traditional practices and modern neuroscience, has demonstrated efficacy in improving motor recovery and reducing long-term disability (Fig. 7). When combined with pharmacological interventions, these strategies can promote synergistic effects, including enhanced neuroplasticity, improved cerebral perfusion, and long-term functional restoration. The integration of traditional modalities with evidence-based pharmacological and rehabilitative approaches, under a multidisciplinary framework, may provide more holistic and sustainable benefits for patients with ICH^94^.

### Personalized prediction and precision medicine

The shift toward personalized medicine in ICH is supported by advances in risk prediction technologies and biomarker-guided stratification (Fig. 7). Population-based cohorts and nomogram models that incorporate variables such as age, BP, and hematoma volume provide refined prognostic insights^95^. Imaging modalities including ASPECTS scoring, dynamic contrast-enhanced CT, permeability MRI, and dual-energy CT offer improved evaluation of hemorrhage risk and BBB status, enabling more individualized therapeutic decisions^96,97^.

BBB integrity is particularly critical in predicting outcomes. Dynamic contrast-enhanced imaging can help guide pharmacological strategies by identifying patients at higher risk of hemorrhagic transformation, thereby balancing reperfusion benefits with safety concerns^98^. Beyond imaging, integration of genetic profiling, clinical history, and molecular biomarkers into predictive models enhances treatment personalization. Artificial intelligence-assisted tools are further improving prediction accuracy, offering real-time support for individualized intervention planning^99^.

### Limitations and translational challenges

Despite substantial progress, the translation of ICH research into consistent clinical benefit faces persistent challenges. Many clinical trials are constrained by small sample sizes and limited patient diversity, with aged and female patients frequently under-represented, thereby limiting generalizability. Heterogeneity in pharmacological regimens, diagnostic criteria, and outcome end points further complicates cross-trial comparisons. Methodological shortcomings, including inadequate randomization, lack of blinding, and over-reliance on observational data, introduce bias and undermine reliability. Preclinical research often overestimates efficacy owing to small cohorts, poorly defined therapeutic windows, and lack of standardized models. To address these limitations, future research must adopt rigorous RCT designs, ensure adequate representation of diverse patient populations, and establish standardized diagnostic and efficacy criteria. Preclinical investigations should emphasize refinement of dosing strategies, therapeutic windows, and cross-validation across models to enhance reproducibility. Bridging these methodological gaps will be essential for generating robust and generalizable evidence, thereby accelerating the translation of promising therapies into clinical practice.

## Conclusion

ICH, particularly spontaneous ICH caused by hypertension in the basal ganglia, remains a leading cause of disability and mortality, with complex pathological mechanisms and multistage progression complicating treatment. As research advances, the management of ICH is evolving from traditional monotherapy to a more comprehensive, multidisciplinary approach, integrating exercise rehabilitation and peripheral–central interactions. This shift is not only based on traditional therapies but also incorporates modern interventions, such as stem cell therapy, which shows promise in promoting neural regeneration, protecting the BBB, and regulating inflammation.

Recent research highlights the importance of early rehabilitation and personalized treatment strategies. Exercise interventions, combined with novel approaches such as neuroprotection, stem cell therapy, and physical modalities, are progressively becoming integral to ICH recovery. Furthermore, the development of precision medicine, focussing on secondary brain injury and therapies targeting ferroptosis, inflammation, and oxidative stress, provides a solid foundation for enhancing treatment outcomes. Despite significant progress, challenges remain in optimizing long-term recovery and managing complications. Future efforts should focus on refining personalized treatment plans using advanced imaging technologies, biomarkers, and dynamic monitoring. Additionally, the validation of new therapies through rigorous clinical trials, optimizing drug delivery methods, and refining treatment timing are crucial to improving clinical outcomes.

In conclusion, the integration of innovative therapies and rehabilitation strategies offers new hope for ICH treatment. Multidisciplinary collaboration and continued exploration of peripheral–central interaction mechanisms will drive improvements in treatment efficacy, ultimately enhancing the quality of life and long-term survival rates for patients with ICH.
